# Spectroscopic evaluation of carcinogenesis in endometrial cancer

**DOI:** 10.1038/s41598-021-88640-7

**Published:** 2021-04-27

**Authors:** Joanna Depciuch, Edyta Barnaś, Joanna Skręt-Magierło, Andrzej Skręt, Ewa Kaznowska, Kornelia Łach, Paweł Jakubczyk, Jozef Cebulski

**Affiliations:** 1 Institute of Nuclear Physics, Polish Academy of Science, 31-342 Krakow, Poland; 2 Institute of Health Sciences, Medical College, University of Rzeszow, Kopisto 2a, 35-959 Rzeszow, Poland; 3 Institute of Medical Sciences, Medical College, University of Rzeszow, Kopisto 2a, 35-959 Rzeszow, Poland; 4 Chair of Morphological Sciences, Department of Pathomorphology, Medical College, University of Rzeszow, Kopisto 2a , 35-959 Rzeszow, Poland; 5 Department of Pediatrics, Institute of Medical Sciences, Medical College, University of Rzeszow, Warzywna 1A, 35-310 Rzeszow, Poland; 6 Institute of Physics, College of Natural Sciences, University of Rzeszow, Pigonia 1, 35-310 Rzeszow, Poland

**Keywords:** Analytical chemistry, Infrared spectroscopy, Medical and clinical diagnostics

## Abstract

Carcinogenesis is a multifaceted process of cancer formation. The transformation of normal cells into cancerous ones may be difficult to determine at a very early stage. Therefore, methods enabling identification of initial changes caused by cancer require novel approaches. Although physical spectroscopic methods such as FT-Raman and Fourier Transform InfraRed (FTIR) are used to detect chemical changes in cancer tissues, their potential has not been investigated with respect to carcinogenesis. The study aimed to evaluate the usefulness of FT-Raman and FTIR spectroscopy as diagnostic methods of endometrial cancer carcinogenesis. The results indicated development of endometrial cancer was accompanied with chemical changes in nucleic acid, amide I and lipids in Raman spectra. FTIR spectra showed that tissues with development of carcinogenesis were characterized by changes in carbohydrates and amides vibrations. Principal component analysis and hierarchical cluster analysis of Raman spectra demonstrated similarity of tissues with cancer cells and lesions considered precursor of cancer (complex atypical hyperplasia), however they differed from the control samples. Pearson correlation test showed correlation between cancer and complex atypical hyperplasia tissues and between non-cancerous tissue samples. The results of the study indicate that Raman spectroscopy is more effective in assessing the development of carcinogenesis in endometrial cancer than FTIR.

## Introduction

Endometrial cancer is commonly divided into oestrogen-dependent (type I) and oestrogen-independent (type II). Type I constituting 80% of all cases usually represents low-grade tumours. While type II including the remaining 20% of all cases has poorer clinical prognosis^1^. Carcinogenesis is a multistage process preceding cancer observed in type II endometrial cancer. Endometrial hyperplasia without and with histological atypia are generally accepted as stages of type I carcinogenesis. These stages have only a potential role in type I endometrial cancer carcinogenesis^2^. Hyperplasia without atypia transforms into cancer only in 1–3% of cases, while hyperplasia with atypia in 29% of cases^3^. Currently, based on microscopic evaluation it is impossible to predict which cases transfer to cancer. Therefore, new methods are required to enable this prediction. Spectroscopy candidates for this role. Specimens for both pathological spectroscopic analysis of stages of endometrial cancer carcinogenesis and tissues adjacent cancer are easily available in curettage^4^. Another aim of spectroscopic analysis of stages preceding endometrial cancer may be to determine a new mechanism operating behind the initiation of carcinogenesis.

Currently, histopathological methods are still the gold standard for cancer cells diagnosis in biopsy material. However, these methods are used in high stage of diseases. These methods are not only invasive and time consuming, but also the sensitivity of histopathological methods depends on the subjective judgement of a pathologist. Therefore, very high false negative (1.0 to 1.5 per 1000 women) and false positive (121.2 per 1000 women) rates, were observed^5,6^. Moreover, hematoxylin and eosin (H&E) dyes were used for histochemical staining of the tissue samples. Unfortunately, these dyes are non-specific for cancer cells^7^. Biomarkers, defined as disease-related molecular changes in tissues or body fluids, are analysed to decrease the percent of false positive rates^8^. However, the methods used for biomarkers marking are expensive. Moreover, lots of biomarkers are nonspecific^9–11^. Therefore, it is very important to find non-invasive and cost-effective tests, which can be used as novel diagnostic methods. Consequently, it is of prime importance to conduct interdisciplinary studies e.g. in physics to develop methods which can be used in diagnostic of cancer diseases^4,12–14^. Some of these methods are Raman and FTIR spectroscopy. They allow chemical characterization of studied samples. Moreover, Raman and FTIR spectroscopy are non-invasive, simple, accurate and rapid. These non-destructive testing methods do not cause any damage to the evaluated materials. Spectroscopy could be used as a comparative for gold standard methods facilitating clinical decision-making and patient outcomes by detecting biochemical changes in tissues before the changes can be observed under microscope. Nowadays, Raman and FTIR spectroscopy methods are used to detect chemical changes caused by lung^12,15^, bones^16,17^, thyroid^13,18^, breast^19^, prostate^20^, endometrial cancers^4^. However, recognizing changes visible during neoplastic process is important in the cancer diagnostics. Changes caused in the subsequent stages of cancers should be investigated to understand the neoplastic disease development process. For this purpose, Raman and FTIR spectroscopy can be used to obtain information about chemical changes. Importantly, our previous study demonstrated the potential of FT-Raman and FTIR spectroscopy for the identification of chemical changes in atypical complex hyperplasia and endometrioid adenocarcinoma cancer^4^. To the best of our knowledge, this is the first study applying physical methods such as FT-Raman and FTIR spectroscopy with multidimensional analysis and correlation test to evaluate carcinogenesis in endometrial cancer. The paper also attempts to investigates which spectroscopy method is more effective in recognizing the different stages of carcinogenesis.

## Results

**Figure 1:**
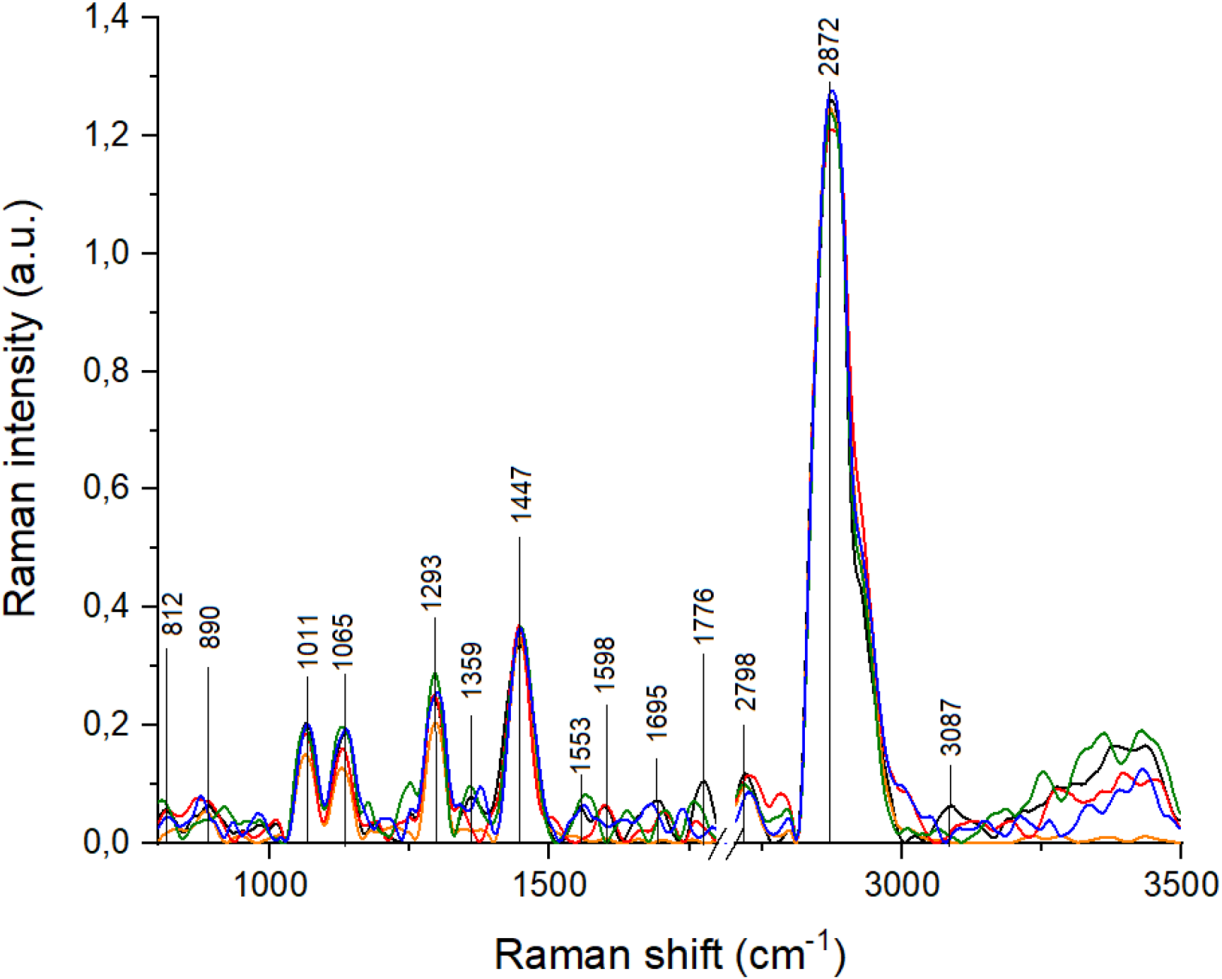
Raman spectra of samples: control (black spectrum); atrophic endometrium (orange spectrum); complex atypical hyperplasia (red spectrum); endometrial polyp (green spectrum); endometrioid adenocarcinoma (blue spectrum).

Raman spectra of all analyzed groups of samples are presented in Fig. 1. Raman spectrum vibrations from nucleic acid (812 cm^−1^, 1065 cm^−1^, 1293 cm^−1^), proline and hydroxyproline (890 cm^−1^), tryptophan (1359 cm^−1^), protein (1553 cm^−1^, 1598 cm^−1^), amide I (1695 cm^−1^), proteins and lipids (1447 cm^−1^) and only lipids (1776 cm^−1^, 2798 cm^−1^, 2872 cm^−1^) and water (3087 cm^−1^) were visible in control. The differences in the presence of Raman shift were noticed in the tissues from other study groups. The absence of OH vibrations were observed in all study groups compared to the control group. Moreover, peaks corresponding to proline, hydroxyproline, tyrosine, PO_2_^−^ stretching of nucleic acids and C=C, tryptophan (protein assignment) disappeared in Raman spectra of tissues from atrophic endometrium and complex atypical hyperplasia groups. Furthermore, lack of peaks at 1011 cm^−1^ and 1598 cm^−1^ was observed also for Raman spectrum of atrophic endometrium and endometrial polyp. Raman shift originating for C–C stretching from proline and hydroxyproline disappeared in the spectrum of endometrial polyp.

Comparison of the study groups and control one revealed the disappearance of peaks and peak shifts. Significant shift of peak corresponding to CH_3_ stretching from lipids was visible in the Raman spectrum of atrophic endometrium. Moreover, the shift of peaks originating from C–C stretching from proline and hydroxyproline, amide I and CH_3_ stretching from lipids, were noticed in the Raman spectrum of complex atypical hyperplasia. In the spectrum of endometrial polyp, a significant shift of peaks corresponding to amide I and C=O stretching vibrations from lipids were observed. The most significant shift of the largest number of peaks was visible in the Raman spectrum of endometrioid adenocarcinoma when compared to the control group. Significant shift of peaks originating from: proline, hydroxyproline, tyrosine, PO_2_^−^ stretching of nucleic acids, C–C stretching from proline and hydroxyproline, phosphodiester groups in nucleic acids, tryptophan, amide I and C=O stretching from lipids, were visible in the Raman spectrum of endometrioid adenocarcinoma. All analyzed Raman peaks and their shifts are presented in Table 1.

**Table 1:** Raman shift with corresponding vibrations described in the Raman spectra from Fig. 1^20–24^.

No.	Control	Atrophic endometrium	Atypical complex hyperplasia	Endometrial polyp	Endometrioid adenocarcinoma	Vibrations
1	812			806	**821**	Proline, hydroxyproline, tyrosine, PO_2_^−^ stretching from nucleic acids
2	890	884	**867**		**876**	C–C stretching from proline and hydroxyproline
3	1011		1010			Stretching vibrations of CO, CC, OCH from ring of polysaccharides and pectin
4	1065	1064	1064	1061	1067	Proline
5	1293	1296	**1300**	1295	**1302**	Amide III (collagen assignment)
6	1359			1358	**1376**	Tryptophan
7	1447	1447	1446	1448	1447	CH_2_ bending from lipids and proteins
8	1553			1564	1561	C=C, tryptophan (protein assignment)
9	1598		1599			C=N and C=C stretching from protein
10	1695		**1705**	**1638**	**1685**	Amide I
11	1776		1763	**1757**	**1792**	C=O stretching from lipids
12	2798	**2720**	**2782**	2794	2795	CH_3_ stretching from lipids
13	2872	2869	2872	2871	2873	CH_2_ stretching from lipids
14	3087					OH vibrations from water

Bold means statistically significant shift.

**Figure 2:**
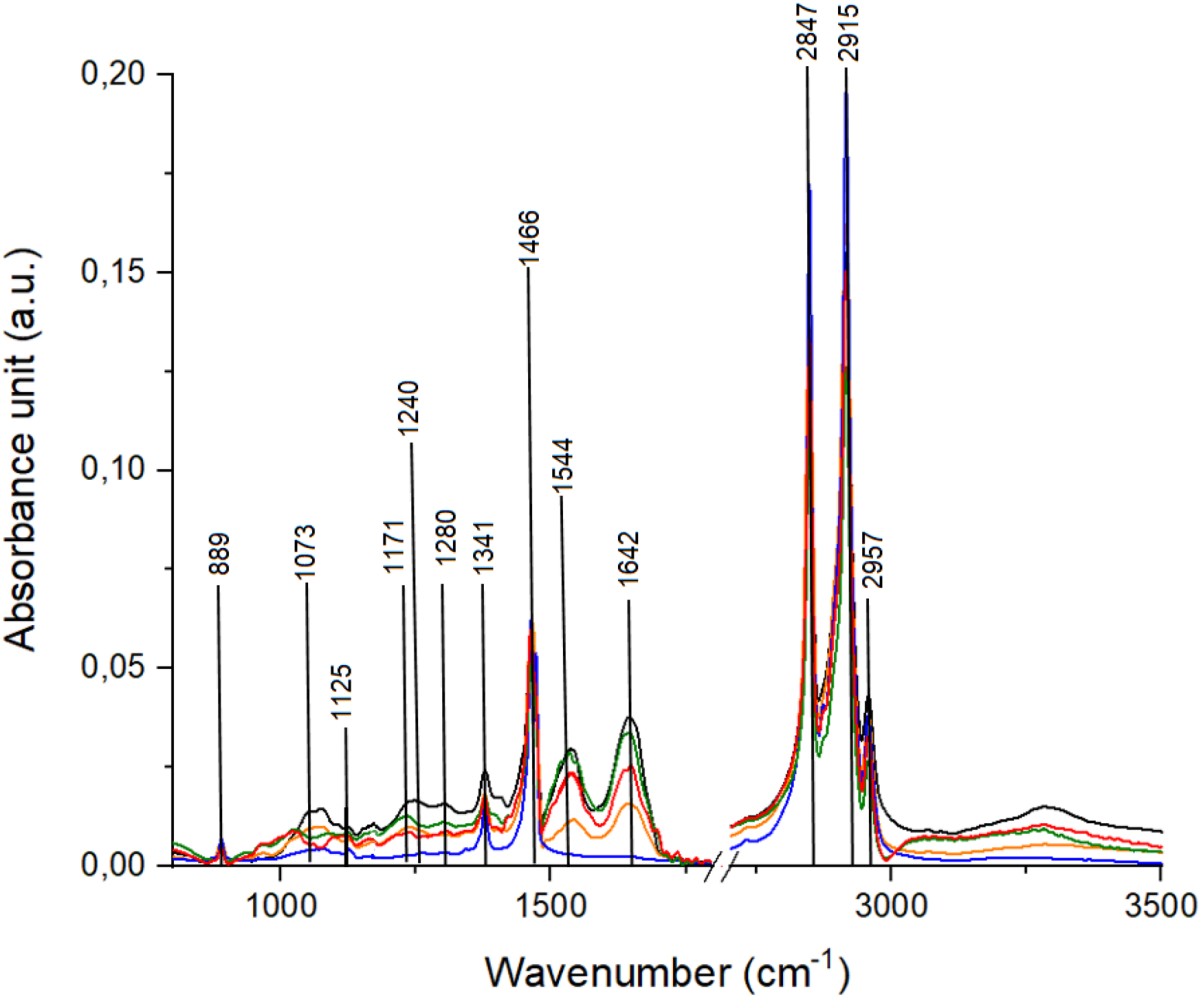
FTIR spectra of samples: control (black spectrum); atrophic endometrium (orange spectrum); complex atypical hyperplasia (red spectrum); endometrial polyp (green spectrum); endometrioid adenocarcinoma (blue spectrum).

FTIR spectra of all analyzed groups of samples are presented in Fig. 2 which demonstrated that all study group samples differed from the control one. In FTIR spectrum of atrophic endometrium (orange plot), significant shift of peaks corresponding to amide II, amide I and CH_2_ as well as CH_3_ groups from lipids vibrations was noticed. Moreover, lack of amide III vibrations and presence of C–O carbohydrates group was observed in comparison with the control group samples. FTIR spectrum of complex atypical hyperplasia (red plot) was characterized by shifting of peaks originating from C–O stretching mode of serine, threonine, and tyrosine, C–O deformation vibrations of proteins, glycogen, carbohydrates, all three amides modes and N–H vibrations of cytosine. The absence of CH_2_ wagging for proline (amino acids and collagen) was also noticed. Comparison of FTIR spectrum of endometrial polyp (red plot) with FTIR spectrum of control group samples (black plot) revealed the presence of C–O group from carbohydrates and absence of C–O stretching mode from serine, threonine, and tyrosine of protein and N–H group of cytosine. Moreover, significant shift of peaks at 1168 cm^−1^, 1245 cm^−1^, 1560 cm^−1^, 2842 cm^−1^ was observed. Finally, in FTIR spectrum of endometrioid adenocarcinoma (blue plot), shift of peaks corresponding to C–O stretching mode of C–OH groups from serine, threonine, tyrosine, amide III, amide II, amide I and absence of deformation N–H cytosine group was visible in comparison to FTIR spectrum of the control group. Description of analyzed peaks present on the FTIR spectra was presented in Table 2.

**Table 2:** FTIR wavenumbers with corresponding vibrations described in the FTIR spectra presented in Fig. 2^25–30^.

No.	Control	Atrophic endometrium	Atypical complex hyperplasia	Endometrial polyp	Endometrioid adenocarcinoma	Vibrations
1	889	888	889	887	889	C–C, C–O deoxyribose,fatty acid, saccharide
2	1073	1078	**1080**	*	**1066**	C–O stretching mode of C–OH groups of serine, threonine, and tyrosine of protein
3	*	**1125**	**1124**	**1124**	*	νC–O Carbohydrates
4	1171	1184	**1167**	**1168**	1169	ν(C–O), ν(C–C), def. C–O–H (proteins, glycogen, carbohydrates)
5	1240	*	**1237**	**1245**	1241	Amide III (N–H bending, C–N stretch, C–C stretch) (proteins, DNA, phospholipids)
6	1280	*	**1304**	*	*	Deformation N–H cytosine
7	1341	1341	*	1341	1340	CH_2_ wagging for proline (amino acids and collagen)
8	1466	1466	**1462**	1465	**1462**	CH_2_ group scissoring modes
9	1544	**1558**	**1535**	**1560**	**1535**	Amide II due to N–H bending and C–N stretching of proteins
10	1642	**1645**	**1646**	1642	**1649**	Amide I (ν(C=O), ν(CN), γ(CCN), δ(NH)) (proteins)
11	2847	**2843**	2847	**2842**	2848	Symmetric stretching of the CH_2_ group due to mainly lipids, with little contribution from proteins, carbohydrates and nucleic acids
12	2915	**2912**	2915	2915	2915	CH_2_ asymmetric stretch: mainly lipids, with little contribution from proteins, carbohydrates, nucleic acids
13	2957	**2953**	2956	2957	2956	CH_3_ asymmetric stretch: mainly lipids

Bold means statistically significant shift.

**Figure 3:**
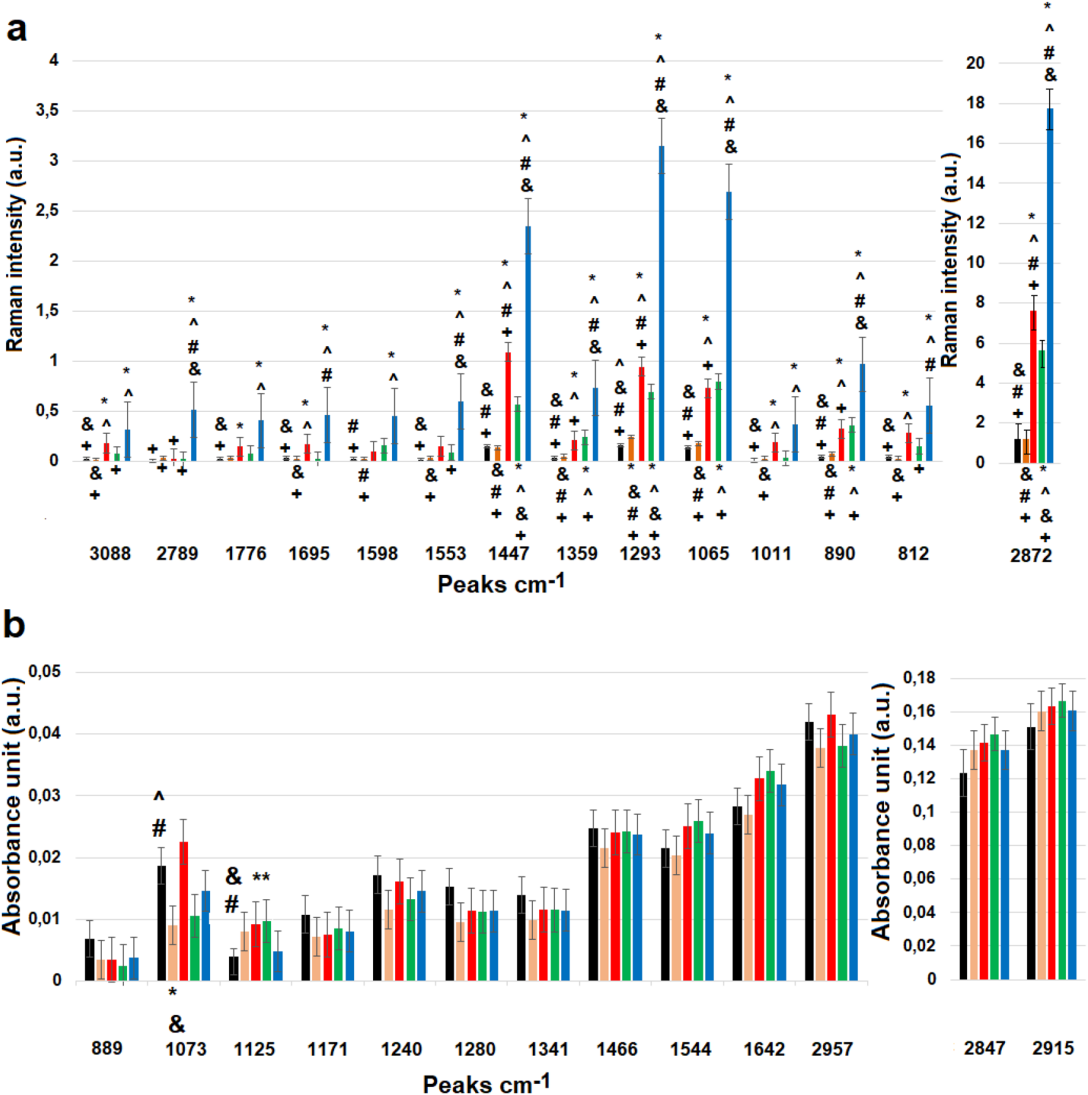
Mean ± standard error of the mean (SEM) intensity values of the individual peaks measured with Raman (**a**) and FTIR (**b**) for all groups of tissue samples, where color bars stand for: control (black spectrum); atrophic endometrium (orange spectrum); complex atypical hyperplasia (red spectrum); endometrial polyp (green spectrum); endometrioid adenocarcinoma (blue spectrum). Data was analyzed using one-way ANOVA followed by Tukey's post hoc test. Statistical significance was adopted at *p < 0.05 versus Control; ^ *p* < 0.05 versus atrophic endometrium; & *p* < 0.05 versus complex atypical hyperplasia; # *p* < 0.05 versus endometrial polyp; + *p* < 0.05 versus endometrioid adenocarcinoma.

Differences in the values of absorbance and Raman intensity were visible in the Figs. 1 and 2. Therefore, average values of these parameters were calculated for all analyzed group of tissue samples, Fig. 3.

Figure 3 presents mean ± SEM intensity values of peaks from the Raman, Fig. 3a, and FTIR, Fig. 3b, spectra. Only statistically significant differences were considered. Figure 3a demonstrated differences between control and atrophic endometrium samples in the 1293 cm^−1^ peak, while differences between control and complex atypical hyperplasia samples were visible at 812 cm^−1^, 890 cm^−1^, 1011 cm^−1^, 1065 cm^−1^, 1293 cm^−1^, 1359 cm^−1^, 1447 cm^−1^, 1695 cm^−1^, 1776 cm^−1^, 2872 cm^−1^ and 3088 cm^−1^ Raman shifts. Differences between control and polyp tissue samples were visible at 890 cm^−1^, 1065 cm^−1^, 129 cm^−1^ 3, 1359 cm^−1^ and 1447 cm^−1^. Moreover, control samples differed from cancer ones in all analyzed Raman spectra. Comparison of atrophic endometrium with complex atypical hyperplasia samples showed no differences at the 1598 cm^−1^, 1776 cm^−1^, 2789 cm^−1^. Atrophic endometrium sample differed from polyp one at the 890 cm^−1^, 1065 cm^−1^, 1293 cm^−1^, 1359 cm^−1^, 1447 cm^−1^, 1598 cm^−1^ Raman shifts, while comparison of this sample with cancer one showed differences in all analyzed spectra. Furthermore, complex atypical hyperplasia tissue samples differed from endometrial polyp ones at the 2872 cm^−1^, 1293 cm^−1^ and 1447 cm^−1^, while with the endometrioid adenocarcinoma, complex atypical hyperplasia tissues differed at 890 cm^−1^, 1065 cm^−1^, 1293 cm^−1^, 1359 cm^−1^, 1447 cm^−1^, 2872 cm^−1^, 2789. When compared to cancer samples, polyp tissue ones did not show any differences at the 1011 cm^−1^ and 1598 cm^−1^. Figure 3b demonstrated, that statistically significant differences between groups were observed only in the case of two analyzed peaks: 1073 cm^−1^ and 1125 cm^−1^. These differences were visible when we compared control and atrophic endometrium, endometrial polyp and control and complex atypical hyperplasia, endometrial polyp. Moreover, statistically significant differences between atrophic endometrium and complex atypical hyperplasia were noticed for the peak at 1073 cm^−1^. In case of the peak at 1125 cm^−1^, statistically significant differences were observed between endometrial polyp, complex atypical hyperplasia samples and the control ones.

**Figure 4:**
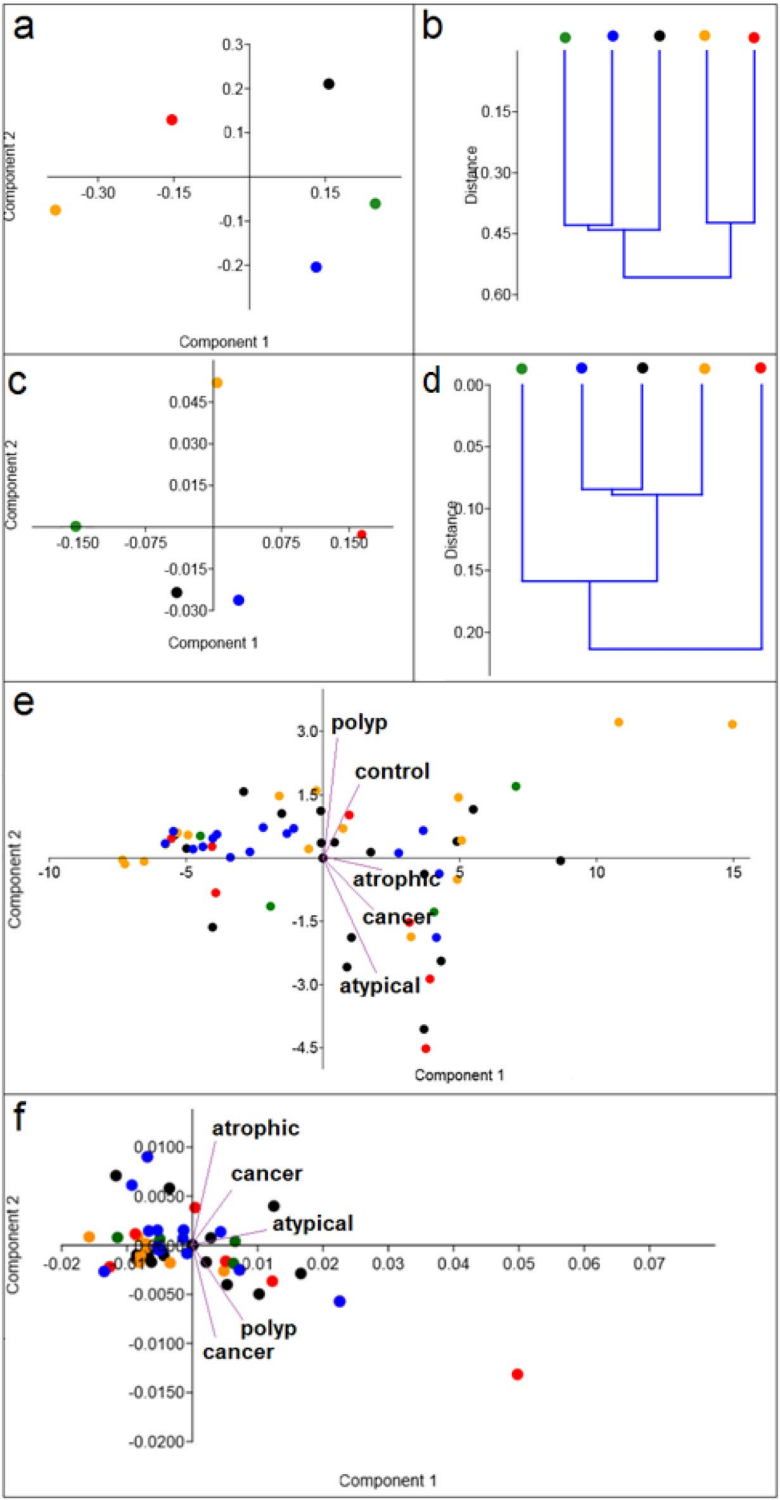
PCA (**a**, **c**, **e**, **f**) and HCA (**b**, **d**) analysis of: control (black dot); atrophic endometrium (orange dot); complex atypical hyperplasia (red dot); endometrial polyp (green dot); endometrioid adenocarcinoma (blue dot) Raman (**a**, **b**, **e**) and FTIR (**c**, **d**, **f**) spectra. Two-dimensional (2D) scores plot of samples with differences in chemical compositions presented through the selected spectral regions: all spectral region for Raman spectra and points corresponding to C–O stretching mode of C–OH groups of serine, threonine, and tyrosine of protein and νC–O vibration from carbohydrates for FTIR data. The results for average spectra were shown in Figure (**a**–**d**) and for all analyzed samples—**e**, **f** with marker with vectors of least-squared lines representing the various groups.

FT-Raman and FTIR spectra variation among the types of samples and their similarity were analysed with principal component analysis (PCA) and hierarchical cluster analysis (HCA), Fig. 4. All analyzed Raman spectra peaks were statistically significant, while in the case of FTIR spectra, only two peaks turned out to be statistically significant. Therefore, PCA analyses were performed only for statistically significant data, which can be further used to differentiate various types of endometrial tissue samples.

**Figure 5:**
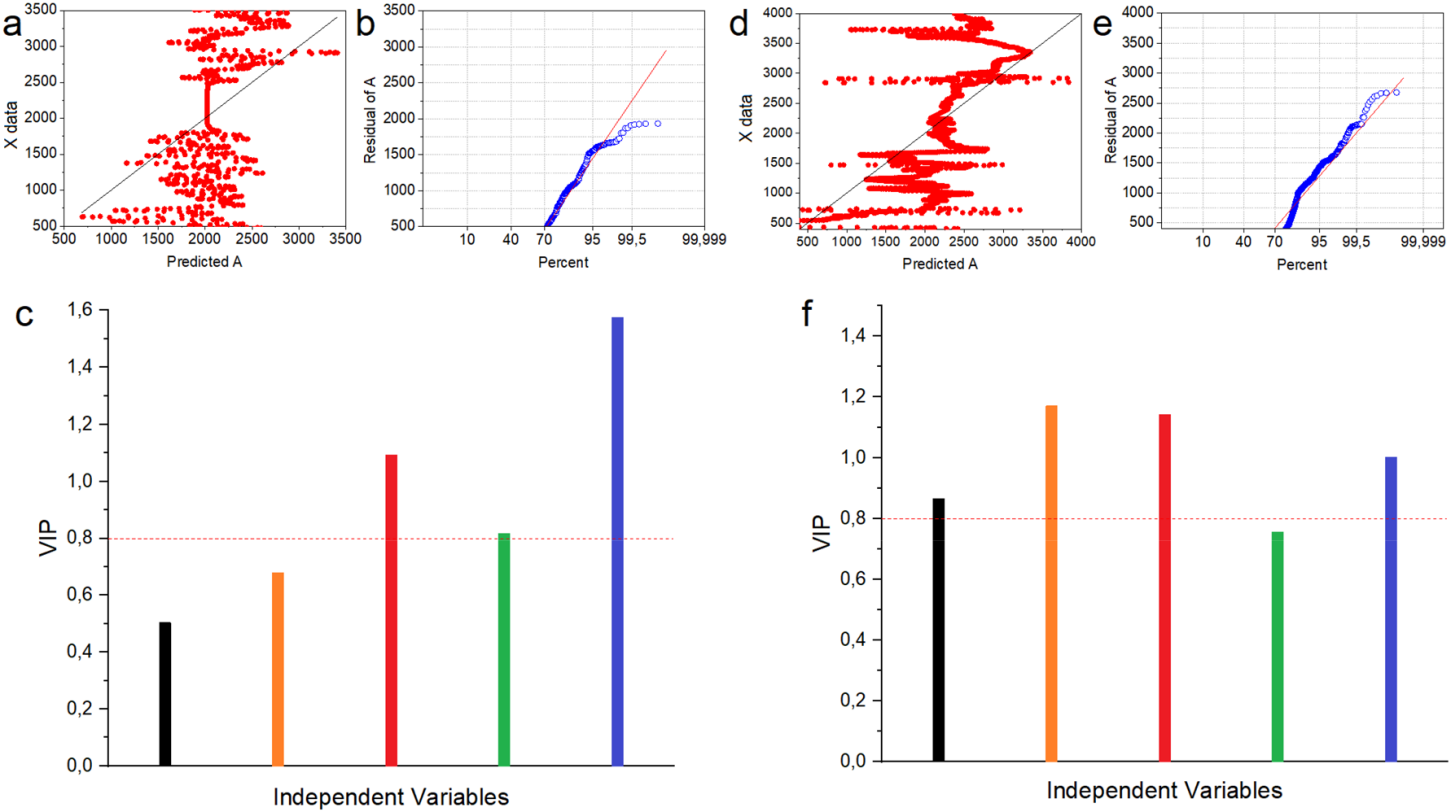
Plots of predicted for Raman (**a**) and FTIR (**d**) region, which could be used to separate endometrial tissues with different carcinogenesis stages. Normal Probability Plot of residual collected from predicted plots for Raman (**b**) and FTIR (**e**) spectra. VIP values plot for the all analyzed Raman (**c**) and FTIR (**f**) range with 0.8 VIP thresholds, where colors stands for the following samples: control (black); atrophic endometrium (orange); complex atypical hyperplasia (red); endometrial polyp (green); endometrioid adenocarcinoma (blue).

PCA analysis of average spectra (Fig. 4a) showed, that Raman spectra of atrophic endometrium, atypical complex hyperplasia, endometrial polyp, endometrioid adenocarcinoma groups differed from the control group. However, similarities in the Raman spectra of endometrial polyp and endometrioid adenocarcinoma groups, were visible. These observations conformed with HCA analysis (Fig. 4b), which showed similarity between these two types of samples. Moreover, HCA analysis also showed that the Raman spectrum of atrophic endometrium group was similar to the one of complex atypical hyperplasia. PCA analysis of average FTIR spectra (Fig. 4c) demonstrated that FTIR spectra of complex atypical hyperplasia and endometrioid adenocarcinoma were placed in the same quarter of the coordinate system. This analysis revealed also similarity between control and endometrial polyp group. Furthermore, HCA analysis of average FTIR spectra (Fig. 4d) showed that control, atrophic endometrium and endometrioid adenocarcinoma create one group which is similar to FTIR spectrum of endometrial polyp tissues. Moreover, PCA of Raman data of all samples, Fig. 4e, revealed that the largest points in the same quarter of the coordinate system referred to endometrioid adenocarcinoma tissue group. However, the highest dispersion was found for atrophic endometrium tissue group. Similar case occurred in the PCA plot obtained for one selected FTIR region, Fig. 4f. Also in this case, significant separation between analyzed group of samples was not observed. Therefore, we marked vectors of least-squared lines representing different groups in Fig. 4e,f. Consequently, PCA obtained from Raman data showed, that atrophic endometrium, complex atypical hyperplasia and endometrioid adenocarcinoma, were separated from control and polyp endometrial tissues. PCA obtained from FTIR data, showed separation between control and polyp, as well as cancer tissue groups. Therefore, Partial Least Squares analysis (PLS) with variables importance in projection (VIP) was performed, Fig. 5.

The PLS results presented as plots of predictor showed, that in the Raman spectra, the region which could be used to separate endometrial tissues with different carcinogenesis stages was between 500 and 1500 cm^−1^ and around 2800–2900 cm^−1^, Fig. 5a. Moreover, the region between 1500 and 1700 cm^−1^, which is very important in description of changes in proteins, can give false positive or false negative results. Obtained prediction correlation was characterized by good predicted response for training data, which was visible in linear fit presented in Fig. 5b. FTIR data analysed with PLS showed good linear fit, Fig. 5e for data enabling separation between each analysed sample in the range between 500 and 1500 cm^−1^ and in the wavenumbers corresponding to CH_2_ and CH_3_ vibrations of lipids, Fig. 5d. VIP values were generated from the model as shown in Fig. 5c,f for Raman and FTIR data, respectively. The threshold level was established at 0.8. Note that the VIP values were not associated for all the measured Raman and FTIR spectra. For the Raman data, VIP values showed that separation of the control and atrophic endometrium samples was impossible, while VIP values obtained for FTIR spectra showed, that polyp samples could not be separated from others. Furthermore, Random Forest as well as second learning machine method C5.0 classification model were calculated to obtain information about accuracy of Raman and FTIR spectroscopy in distinguishing analyzed samples. These two analyses were done for fingerprint FTIR and Raman region (800–1800 cm^−1^), and for a selected region (only peaks which differences were statistically significant—data taken from Fig. 3). The results were presented in Table 3.

**Table 3:** Percentage accuracy with error value of selected and for fingerprint region (800–1800 cm^−1^) for obtained Raman and FTIR spectra.

	Random forest		C5.0	
	Accuracy (%)	Error (%)	Accuracy (%)	Error (%)
Raman selected	92.73	7.27	76.36	23.64
Raman fingerprint	92.73	7.27	83.64	16.36
FTIR selected	96.61	3.39	62.71	37.29
FTIR fingerprint	96.61	3.39	84.75	15.25

The results obtained using Random forest and C5.0 algorithms indicate that both Raman and FTIR approaches can effectively identify groups of cases. The classification accuracy is in the range of 62.71 to 96.61%. It can be seen that much better results were obtained using Random forest algorithm which is an ensemble machine learning method. At the same time, the obtained results provide a basis for further extensive research on a larger number of learning instances.

The correlation between all measured samples was investigated using Pearson correlation test, Table 4.

**Table 4:** Pearson correlation (*p* < 0.05) test for Raman and FTIR spectra.

	Control	Atrophic endometrium	Atypical hyperplasia	Endometrial polyp	Endometrioid adenocarcinoma
**FT-Raman spectroscopy**					
Control		0.80*		0.86*	
Atrophic endometrium	0.80*		0.75*		
Atypical hyperplasia				0.95*	
Endometrial polyp	0.86*	0.97*	0.95*		
Endometrioid adenocarcinoma		0.93*	0.97*	0.93*	
**FTIR spectroscopy**					
Control		0.95*	0.93*	0.95*	0.98*
Atrophic endometrium	0.95*		0.97*	0.84*	0.98*
Atypical hyperplasia	0.93*	0.97*		0.81*	0.96*
Endometrial polyp	0.95*	0.84*	0.81*		0.90*
Endometrioid adenocarcinoma	0.98*	0.98*	0.96*	0.90*	

*Statistically significant, when *p* < 0.05.

The Pearson correlation test obtained from Raman spectra and presented in Table 3 showed, that it was possible to determine the correlation between control, atrophic endometrium and endometrial polyp tissues. Moreover, correlation between atrophic endometrium, endometrial polyp and endometrioid adenocarcinoma was also observed in Raman spectra. Furthermore, correlation in Raman spectra between polyp, control, atypical hyperplasia and endometrioid adenocarcinoma was noticed. Interestingly, lack of correlation between endometrioid adenocarcinoma and other analyzed tissues was visible in Raman spectra. Pearson correlation test obtained from FTIR spectra (Table 3) showed, that each analyzed sample correlated with all others, e.g. correlation between control, atrophic endometrium, atypical hyperplasia, endometrial polyp and endometrioid adenocarcinoma was observed.

## Discussion

Observation of lesions characteristic for cancer cells without significant architectural changes in tissues is very important to understand the carcinogenesis of endometrial cancer. However, the diagnostic gold standard used currently does not allow for it. Therefore, a new diagnostic technique is required to facilitate this application in future. Consequently, this study showed chemical changes that occur during carcinogenesis process in endometrial tissues using FT-Raman and FTIR spectroscopy.

Recent studies show, that all spectroscopic techniques can be used to observe chemical differences between healthy and non-healthy endometrial tissues^4,31–36^. Patel et al. showed, that amides and prolines vibrations can be used as spectroscopic marker in endometrial cancer^31^. Our Raman results also showed, that quantitative and qualitative changes in endometrial cancer tissues are observed at 1695 cm^−1^ and 812 cm^−1^ wavenumbers, which corresponded to amide I and proline vibrations, respectively, Fig. 1. Moreover, we also noticed structural changes in amides vibrations in FTIR spectra, Fig. 2. Similar results were obtained by Taylor et al.^37^. They used FTIR spectroscopy to identify chemical changes that occur at different stages of endometrial cancer. Furthermore, the changes in amides vibrations are more significant, when the cancer stage in higher. As shown in Table 2 shifts were observed in amides vibrations. Interestingly, in comparison with control samples, these shifts were more significant for samples with more developed carcinogenesis process. It is important, especially, when attempting to differentiate complex atypical hyperplasia and endometrial cancer, because differential diagnosis between these two types of endometrial tissues still a problem^38^. Some research showed, that p53 protein can be a factor, which could be used in diagnostics of these two endometrial changes^39^. Therefore, results obtained from the Raman and FTIR range corresponding to amides vibrations confirmed the molecular biology hypothesis. Moreover, the differences in the proteins region in spectra can suggest, that expression of genes and, consequently, proteins conformation could be used as a biomarker in the different stage of endometrial carcinogenesis.

Paraskevaidi et al. used blood serum collected from women suffering from complex atypical hyperplasia and endometrial cancer stage I and II^36^. We observed in FTIR spectra of analysed samples that chemical changes visible in tissue samples corresponded with these noticed in blood serum, especially in amides IR range. However, Paraskevaidi et al. showed also that CH_2_ wagging vibrations from collagen played important role in carcinogenesis process detected from blood serum. In our study significant shift of collagen vibrations was visible in Raman spectra of the samples characterized by the most advanced carcinogenesis process, Fig. 1, Table 2. Moreover, intensity of peaks originating from functional groups building collagen structure decrease together with carcinogenesis process. It could be caused by collagen linearization and tissue stiffness and such changes increase the tumor incidence and progression^40^. Collagen constitutes the scaffold of tumor microenvironment and regulating its extracellular matrix remodeled by collagen degradation and re-deposition, and promoting tumor infiltration, angiogenesis, invasion and migration^41^. Therefore, we think, that also vibrations of collagen functional groups can be used as spectroscopic marker of carcinogenesis.

Moreover, PCA analysis of average spectra of different types of tissues, showed, that only in the case of Raman data, differentiation between cancer and complex atypical hyperplasia was possible, Fig. 4a. These two kinds of endometrial changes differed enough to allow differentiation^38^. The histopathology image of complex atypical hyperplasia is very similar to the one of cancer. Therefore, new methods are required that enable differentiation of these two kinds of endometrial changes. However, PCA analysis of all analyzed samples measured with Raman (Fig. 4a) and FTIR (Fig. 4b) did not show separation between each stage of carcinogenesis. The similarity between control and polyp endometrial tissues, was detected from the Raman data, while PCA analysis of FTIR spectra showed non-separation between control, atrophic endometrium and complex atypical hyperplasia. Polyp and atrophic endometrium are classified as normal endometrial tissues without any signs of neoplastic changes^42^. Complex atypical hyperplasia is very similar to the image of cancer^38^. Therefore, FTIR may not be used to separate very similar carcinogenesis stages. However, in this study we used sample collected from women at different age and it is known that age greatly influences FTIR and Raman spectra obtained^43^.

As shown in Figs. 1, 2 and 3 endometrial tissues in different carcinogenesis differs fundamentally from normal tissue in terms of structure, genetics, and cellular activity. Therefore, obtained spectra were analyzed by multivariate analysis methods^44,45^. In this study we performed PCA analysis and Person correlation test, Fig. 3, Table 3, respectively. PCA analysis, as well as Person correlation test demonstrated that Raman spectroscopy offers higher possibility to distinguish between analysed types of tissues. The reason could be the difference in the physical principles of both techniques^46^, e.g. FTIR spectroscopy is sensitive especially for OH stretching in water, while Raman spectroscopy to—C–C, C=C, and C≡C bonds^47^. A high number of carbon functional groups was observed in biological materials, therefore Raman spectroscopy can give a more precise chemical composition of a sample. Particularly, in Fig. 3 the differences in the peak area were observed for almost all analyzed Raman peaks, and only for two FTIR peaks, which corresponded with functional groups building carbohydrates and protein amino acid. These suggest, that changes observed by us using two complementary methods, which have the greatest impact on endometrial carcinogenesis occur in carbohydrates and amino acids, thus consequently in protein. Indeed, lectins, which are carbohydrate-binding proteins permit glycoproteins to be expressed on cancer cells^48^. Furthermore, other research showed, that in the cancer cells there are changes in the expression of genes that code glycoproteins^49^. Importantly, the changes in the carbohydrates and amino acids metabolism and expression, were visible not only in the case of endometrial carcinogenesis, but also in other types of cancer, e.g. colon^50^, overian^51^ ones etc.”

## Conclusions

In this study, Raman and FTIR spectroscopy were used to evaluate a carcinogenesis in endometrial cancer. Moreover, the methods were compared in terms of efficiency in detection of chemical changes during carcinogenesis of endometrial cancer. Obtained Raman spectra showed, that together with endometrial cancer development, chemical structure and compositions of tissues differs from the ones observed in control samples. Moreover, these changes are statistically significant in all analyzed Raman ranges, while only two peaks of values of absorbance in the FTIR spectra were statistically significant (1073 cm^−1^ and 1125 cm^−1^). Furthermore, PCA and HCA analysis of Raman spectra showed that tissues with cancer cells or changes which occurred before cancer (complex atypical hyperplasia) are similar to each other, but they are different from the control samples or changes which can be observed in non-cancerous endometrial tissues. Correlation was found between non-cancerous tissues, as well as between cancer and complex atypical hyperplasia tissues in the correlation test. In contrast, PCA and HCA results as well as correlation obtained from FTIR data showed, that samples which are not histologically similar, correlated and were characterized by similar chemical changes. Overall, our results indicate that Raman spectroscopy is more effective than FTIR in assessing the development of carcinogenesis in endometrial cancer. Using Raman spectroscopy helps to investigate information about atrophic endometrium, complex atypical hyperplasia and endometrioid adenocarcinoma development, while FTIR spectroscopy facilitates differentiation between healthy, cancer and polyp endometrial tissues. Consequently, FTIR spectroscopy methods can be used to detect more visible changes in pathomorphologic images, but not in the case of very similar carcinogenesis phase. Importantly, the amount of tissues collected from the women at different age investigated in this study was not extensive. Therefore, further tests should be performed to confirm the obtained results. Moreover, the results need to be verified by other researchers. Moreover, application of Raman and FTIR spectroscopies as diagnostics tools in carcinogenesis process should be studied regarding other organs.

## Materials and methods

### Materials limitation

The study was conducted under Institutional Review Board (No. 9/05/2019) at Rzeszow University. The experimental protocols used in this study were approved by the institutional ethics committees (IECs) of the University of Rzeszow, and were carried out in accordance with the approved guidelines. Informed consents were obtained from all subjects. All studied material (59 tissues samples) was obtained from the women treated in Podkarpackie Gynecology and Obstetrics Ward, Oncology Center in Brzozow, Gynecology and Obstetrics Clinic of the Provincial Clinical Hospital No. 1 in Rzeszow and the Municipal Hospital in Rzeszow. All samples were divided into 5 groups: control (17 tissues); atrophic endometrium (12 tissues); complex atypical hyperplasia (8 tissues); endometrial polyp (6 tissues); endometrioid adenocarcinoma (16 tissues). The medical characteristics of patients, from whom we obtained samples, is presented in Table S1. Moreover, particularly important is the issue that samples were collected from 16 patients over several years, where each next sample were characterized by other endometrial changes. Furthermore, noteworthy is relatively small number of tissue samples in overall group and distribution of their subgroups. It is connected with character of the Clinic and does not reflect their frequency in population.

### Materials preparation

All obtained materials were prepared like in the paper Depciuch et al.^52^. First, tissues were placed for 12 h in a liquid fixative. Secondly, ethanol within the tissue was gradually replaced with xylene. A paraffin infiltration of the tissue was performed at a temperature of 52 °C. When infiltration sections were embedded in paraffin block, they were prepared by pouring liquefied paraffin into a metal mold and thin piece of tissue was inserted with the appropriate spatial orientation. Each section was flattened on a hot water surface. For the spectroscopic measurements, the obtained samples were placed on CaF_2_ slides. Moreover, an immunohistochemical diagnostics was performed for each obtained sample.

## Methods

All methods were performed in accordance with the relevant guidelines and regulations.

### FT-Raman measurement

For the FT-Raman spectra Nicolet NXR 9650 FT-Raman Spectrometer was used. The spectrometer has an Nd:YAG laser (1064 nm) and a germanium detector. The measurement range was between 150 and 3.700 cm^−1^. The laser power was 0.5 W. Moreover, each sample was measured using 64 scans with 8 cm^−1^ resolution. All obtained spectra were analyzed by OPUS software using baseline correction, smoothing (7 points) and normalization using vector normalization functions.

### FTIR measurements

Vertex 70v spectrometer by Bruker was used to obtain FTIR spectra of all analyzed samples,. Moreover, Attenual Total Reflectance (ATR) technique was used with diamond crystal. All samples were measured in the IR range between 400 and 4000 cm^−1^ using 32 scans with 2 cm^−1^ resolutions. Moreover, all measurements were made in triplicate. The obtained spectra were normalized, smoothed and baseline corrections were made in the OPUS software.

### Statistics—multivariate analysis, Pearson correlation test

Principal component analysis (PCA) was performed to obtain information about the spectra variation among the types of samples. PCA reduced the dimensionality, the number of variables of the data, while maintaining as much variance as possible. PCA was performed based on the selected spectral regions, which were determined after counting the average values of Raman intensities and absorbance of analyzed peaks. The statistical significance of the calculated values of Raman intensities and absorbance were analyzed by one-way ANOVA followed by the Tukey's test (Statistica 10). The obtained experimental results were represented as the means ± SEM (the standard error of the mean). Furthermore, HCA analysis was performed to determine similarity between each group of samples. Moreover, to obtain information about correlation between obtained Raman and FTIR spectra of measured samples, the Pearson’s test was performed with *p* < 0.05 and significance level of 95%. All analyses were performed using Past 3.0 software. In addition, considering low number of samples in this study, Partial Least Square (PLS) analysis was performed. It was used in case of multicollinearity problems associated with complex biological data when the number of predictors was much larger that the number of samples in tall or wide data sets, like in this study. Moreover, Variables importance in projection (VIP) was calculated to define the most important vibrational band associated with the separation between each type of measured samples. The PLS analysis and VIP factor were calculated using Origin 2019 software. Random forest as well as second learning machine method C5.0 classification model were calculated to obtain information about accuracy of Raman and FTIR spectroscopy in distinguishing of analyzed samples. We used the suggested Random forest algorithm^53^ and additionally applied the traditional C5.0 single decision tree algorithm well known in the literature^54^. The R environment and the Random Forest and C5.0 software packages were used to conduct the experiments.

## Supplementary information

Supplementary Informations.
