# Supplementary material for: Spectroscopic evaluation of carcinogenesis in endometrial cancer
Source: Sci Rep. 2021 Apr 27;11:9079. doi: 10.1038/s41598-021-88640-7 (PMC8079695; doi:10.1038/s41598-021-88640-7)
Supplement: Supplementary file 1 — Supplementary Informations. [file 41598_2021_88640_MOESM1_ESM.docx]

**Spectroscopic evaluation of carcinogenesis in endometrial cancer**

**Joanna Depciuch^1, *, #^, Edyta Barnas^2,*^, Joanna Skręt-Magierło^3,*^, Andrzej Skręt^3^, Ewa Kaznowska^4^, Kornelia Łach^5^, Paweł Jakubczyk^6^, Józef Cebulski^6^**

^1^ Institute of Nuclear Physics, Polish Academy of Science, 31-342 Krakow, Poland

^2^ Institute of Health Sciences, Medical College, University of Rzeszow, Kopisto 2a, 35-959 Rzeszow, Poland

^3^ Institute of Medical Sciences, Medical College, University of RzeszowKopisto 2a, 35-959 Rzeszow, Poland

^4^ Department of Pathomorphology, Chair of Morphological Sciences, Medical College, University of Rzeszow, Kopisto 2a, 35-959 Rzeszow, Poland

^5^ Clinic of Pediatric Oncology and Hematology, College of Medical Sciences, University of Rzeszow, Kopisto 2a, 35-959 Rzeszow, Poland

^6^  Institute of Physics, College of Natural Sciences, University of Rzeszow, Pigonia 1, 35-310 Rzeszow,Poland

^*^equal first author

^#^corresponding author: [joanna.depciuch@ifj.edu.pl](mailto:joanna.depciuch@ifj.edu.pl)

| **No** | **Age** | **1st date of collection** | **Histopalogical diagnosis 1** | **2nd date of collection** | **Histopalogical diagnosis 2** | **3rd date of collection** | **1st histopatological cancer type** | **grading** | **image of residual endometrium** | **4th date of collection** | **2 nd histopatological cancer type** | **grading2** | **pTNM** | **image of residual endometrium** |
| --- | --- | --- | --- | --- | --- | --- | --- | --- | --- | --- | --- | --- | --- | --- |
| 1. | 36 | 2014 | **1/1**  Dyshormonogenetic endometrium. Endometrial polyp | 2015 | **1/2**  Simplex and complex hyperplasia with focal atypia | 2016 | Endometrioid adenocarcinoma | G1 | Endometrial polyp, atypical complex hyperplasia, pseudodecidual changes after hormonotherapy | 2017 | **1/3** Endometrioid adenocarcinoma | G1 | pT1a | **1/4**  Atypical complex hyperplasia |
| 2. | 55 | 2017 | **2/1** Control | 2017 | **2/2**  Atrophic endometrium | 2018 | Endometrioid adenocarcinoma in situ |  | Endometrial polyp. Atypical complex hyperplasia. | 2018 | **2/3** Endometrioid adenocarcinoma | G1 |  | **2/4** Simplex and complex hyperplasia. |
| 3. | 27 | 2018 | **3/1**  Atypical complex hyperplasia |  |  | 2018 | Endometrioid adenocarcinoma in situ. Invasive endometrioid adenocarcinoma | G1 |  | 2018 | **3/2** Endometrioid adenocarcinoma | G1 | pT1a | **3/3** Atypical complex hyperplasia . |
| 4. | 86 | 2018 | **4/1** Endometrial polyp |  |  | 2018 | Endometrioid adenocarcinoma | G1 | Endometrial polyp. Atypical complex hyperplasia. | 2018 | **4/2** Endometrioid adenocarcinoma | G1 | pT1a | **4/3** Atypical complex hyperplasia. |
| 5. | 65 |  |  |  |  |  |  |  |  | 2017 | **5/2** Endometrioid adenocarcinoma | G2 | pT3b | **5/3** Atrophic endometrium |
| 6. | 71 | 2012 | **6/1**  Control | 2013 | **6/2**  Control |  |  |  |  | 2017 | **6/3** Endometrioid adenocarcinoma | G1 | pT1a | **6/4**  Endometrial polyp |
| 7. | 70 | 2016 | **7/1**  Control | 2017 | **7/2**  Control |  |  |  |  | .2018 | **7/3**  Endometrioid adenocarcinoma | G2 | pT1b | **7/4** Atrophic endometrium |
| 8. | 61 | 2018 | **8/1** Complex hyperplasia |  |  |  |  |  |  | 2018 | **8/2** Endometrioid adenocarcinoma | G1 | pT1a | **8/3** Atrophic endometrium |
| 9. | 82 | 2015 | **9/1** Control | 2016 | **9/2** Control |  |  |  |  | 2018 | **9/3** Endometrioid adenocarcinoma |  | pT3. naciekanie przedsionka pochwy | **9/4** Atrophic endomterium |
| 10. | 63 | 2016 | **10/1**  Control | 2016 | **10/2** Atrophic endometrium | 2017 | **10/3** Endometrioid adenocarcinoma |  | Endometrial polyp | 2018 |  |  |  | **10/4** Atrophic endometrium |
| 11. | 53 | 2012 | **11/1**  Control | 2014 | **11/2** Endometrial polyp |  |  |  |  | 2016 | **11/3** Endometrioid adenocarcinoma | G1 | pT2 | **11/4** Atrophic endometrium |
| 12. | 52 | 2011 | **12/1**  Control | 2013 | **12/2** Control |  |  |  |  | 2016 | **12/3** Endometrioid adenocarcinoma | G2 | pT1a | **12/4** Atrophic endometrium |
| 13. | 60 | 2014 | **13/1** Control | 2015 | **13/2** Control |  | Endometrioid adenocarcinoma Variant with squammous differentiation |  |  | 2016 | **13/3** Endometrioid adenocarcinoma Variant with squammous differentiation | G1 | pT1a | **13/4** Atrophic endometrium |
| 14. | 56 | 2012 | **14/1**  Late secretion phase. Endometrial polyp | 2014 | **14/2**  Endometrial polyp | 2016 | Atypical simplex hyperplasia |  |  | 2016 | **14/3** Endometrioid carcinoma | G1 | pT2 | **14/4.** Atypical complex hyperplasia |
| 15. | 75 | 2015 | **15/1** Control | 2015 | **15/** Control |  |  |  |  | 2016 | **15/3**  Endometrioid carcinoma | G1 | pT2 | **15/4** Atrophic endomterium |
| 16. | 56 | 2016 | **16/1** Control | 2018 | **16/2** Control |  |  |  |  | 2019 | **16/3**  Endometrioid adenocarcinoma. Mucinous adenocarcinoma | G1 | pT1a | **16/4** Atrophic endometrium |

**Table S1. Medical characteristic of patients from which samples were collected.**

**Consent for study participation**

Surname and name ………………………………………………………………………………………….

Biopsy of the breast is performed to collect material for histopathological examination, which will show if there are cancer cells in the entometrial. The type of biopsy performed (e.g. Magnum biopsy, vacuum-assisted biopsy - mammotomy) and how many biopsies (tissue fragments) will be taken depends on the decision of the doctor, after analysis of the radiological documentation and clinical data. Usually there are 4-12 specimens (tissue fragments) taken, which can be used also in scientific research.

After the localisation of the biopsy site, that part of your breast will be anesthetized topically using Xylocaine. A small skin incision (approximately 5 mm) will then be made in the area around the lesion, the specimens will be taken through the cut. The markers are usually made of titanium (3-4 mm in size) and depending on the type, they may additionally contain collagen or carbohydrate component. In case of allergies to metals or other substances, it should be reported before starting the procedure to the staff of the biopsy laboratory.

To avoid complications during and after the biopsy, it is necessary to stop the intake (about 7-10 days before the biopsy) of drugs that inhibit blood clotting (including Aspirin, Acard, Polopyrin, if in doubt, ask your doctor). The patient should inform the doctor performing the biopsy about their blood coagulation disorders, allergies, chronic

diseases (e.g. viral hepatitis, thrombocytopenia). On the day of the biopsy, the patient should visit biopsy room with the current blood clotting tests results (INR, prothrombin time, APTT, fibrinogen).

Due to the patient's safety, the doctor may refrain from performing the biopsy if the patient has not stopped taking medications that inhibit blood clotting, or there are other factors that increase blood clotting – due to the risk of complications.

If the patient reports for a biopsy without the current blood clotting tests results, a biopsy will not be performed on that day. Due to the possibility of post-biopsy complications (e.g. prolonged bleeding, hematoma), the patient remains under control of the biopsy office for about 1 hour (after the MAGNUM biopsy) and about 3 hours after the vacuum-assisted core needle biopsy. Recommendations for the procedures after the biopsy will be provided to the patient together with the information card.

I have read and understood the information on the biopsy above.

Date ……………………… .. Patient's signature ………………………
